# Evaluating the protein coding potential of exonized transposable element sequences

**DOI:** 10.1186/1745-6150-2-31

**Published:** 2007-11-26

**Authors:** Jittima Piriyapongsa, Mark T Rutledge, Sanil Patel, Mark Borodovsky, I King Jordan

**Affiliations:** 1 School of Biology, Georgia Institute of Technology, Atlanta, GA 30332, USA.; 2 Wallace H. Coulter Department of Biomedical Engineering, Georgia Institute of Technology and Emory University, Atlanta, GA 30332, USA.; 3 Division of Computational Science and Engineering at College of Computing, Georgia Institute of Technology, Atlanta, GA 30332, USA.

## Abstract

**Background:**

Transposable element (TE) sequences, once thought to be merely selfish or parasitic members of the genomic community, have been shown to contribute a wide variety of functional sequences to their host genomes. Analysis of complete genome sequences have turned up numerous cases where TE sequences have been incorporated as exons into mRNAs, and it is widely assumed that such 'exonized' TEs encode protein sequences. However, the extent to which TE-derived sequences actually encode proteins is unknown and a matter of some controversy. We have tried to address this outstanding issue from two perspectives: i-by evaluating ascertainment biases related to the search methods used to uncover TE-derived protein coding sequences (CDS) and ii-through a probabilistic codon-frequency based analysis of the protein coding potential of TE-derived exons.

**Results:**

We compared the ability of three classes of sequence similarity search methods to detect TE-derived sequences among data sets of experimentally characterized proteins: 1-a profile-based hidden Markov model (HMM) approach, 2-BLAST methods and 3-RepeatMasker. Profile based methods are more sensitive and more selective than the other methods evaluated. However, the application of profile-based search methods to the detection of TE-derived sequences among well-curated experimentally characterized protein data sets did not turn up many more cases than had been previously detected and nowhere near as many cases as recent genome-wide searches have. We observed that the different search methods used were complementary in the sense that they yielded largely non-overlapping sets of hits and differed in their ability to recover known cases of TE-derived CDS. The probabilistic analysis of TE-derived exon sequences indicates that these sequences have low protein coding potential on average. In particular, non-autonomous TEs that do not encode protein sequences, such as Alu elements, are frequently exonized but unlikely to encode protein sequences.

**Conclusion:**

The exaptation of the numerous TE sequences found in exons as *bona fide *protein coding sequences may prove to be far less common than has been suggested by the analysis of complete genomes. We hypothesize that many exonized TE sequences actually function as post-transcriptional regulators of gene expression, rather than coding sequences, which may act through a variety of double stranded RNA related regulatory pathways. Indeed, their relatively high copy numbers and similarity to sequences dispersed throughout the genome suggests that exonized TE sequences could serve as master regulators with a wide scope of regulatory influence.

**Reviewers:**

This article was reviewed by Itai Yanai, Kateryna D. Makova, Melissa Wilson (nominated by Kateryna D. Makova) and Cedric Feschotte (nominated by John M. Logsdon Jr.).

## Background

Transposable elements (TEs) are DNA sequences capable of moving (transposing) among locations in the genomes of their host organisms. When TEs transpose they often replicate themselves and they can accumulate to very high copy numbers. For instance, at least 47% of the human genome is made up of TE-derived sequences [1]. For many years, TEs were thought to be genomic parasites that did not contribute functionally relevant sequences to the genomes in which they reside [2,3]. However, as of late it has become increasingly apparent that TEs can have profound effects on the structure, function and evolution of their host genomes [4-7].

One way that TEs have contributed to the function and evolution of their host genomes is through the donation of regulatory sequences that control the expression of nearby genes. This phenomenon was originally noticed through the elucidation of individual cases where host genes were found to be regulated by TE-derived sequences [8,9]. Later, genome-scale analyses confirmed that TE-derived sequences have contributed diverse and abundant regulatory sequences to host genomes [10,11].

TEs can also contribute to host genomes by providing protein coding sequences. This process is initiated when a new or existing TE sequence becomes captured as an exon (exonized) in a host gene mRNA sequence. The exonization of TE sequences appears to be quite common in eukaryotic genomes. An early high-throughput analysis of the human transcriptome by Nekrutenko and Li revealed that 4% of human protein coding regions contained TE sequences [12]. However, the extent to which exonized TE sequences actually contribute *bona fide *protein coding sequences has been called into question. It is simply not clear whether the presence of a TE sequence in a spliced exon, *i.e*. as part of an mRNA, indicates that it will ultimately be translated into a functioning protein.

Two reports in particular have challenged the figure of 4% of human proteins with TE-derived coding sequences. In both of these studies, more conservative approaches to the identification of TE-derived protein coding sequences were taken. Specifically, these studies employed the analysis of coding sequences taken exclusively from proteins that had been experimentally characterized, either through elucidation of their 3D structures or via direct peptide sequencing methods. Thus, only the best characterized protein coding sequences were studied and gene predictions, or models, based on the mapping of expressed sequences to genomes were not considered. This approach was first taken by Pavlicek *et al*. who surveyed a dataset of 781 non-redundant human proteins with 3D structures for the presence of TE-derived coding sequences [13]. They were not able to find a single reliable case of a TE-derived protein coding sequence in these data. Considering these results together with the previous work of Nekrutenko and Li [12], the authors concluded that while many alternative transcripts may include TE sequences, these are rarely if ever incorporated into the mRNA sequences that are destined to be translated into proteins. Pavlicek *et al*. found it particularly unlikely that non-coding TEs, such as Alu elements, could evolve to encode proteins after being incorporated into host mRNAs.

Gotea and Makalowski conducted a similar, if further reaching, study by looking for TE-derived sequences in the coding regions of human proteins taken from the Protein Data Bank [14] (3,764) and from the SwissProt [15] collection of directly sequenced human peptides (1,765) [16]. Evaluation of these sequences with the RepeatMasker program [17] uncovered 24 cases of TE-derived protein coding sequences. However, many of these had relatively low sequence similarity scores that were close the RepeatMasker threshold for false-positives. After further evaluation of these cases using a variety of comparative sequence analysis techniques, the authors settled on a figure of 0.1% for the percentage of actual protein coding sequences with TE-derived exons. Incidentally, this figure is in line with the initial analysis of the human genome sequence, which found 47 cases of human protein coding regions with TE-derived sequences, corresponding to ~0.16% of all human genes given the total human gene number count of ~30,000 used at that time [1].

While there can be little doubt that these two aforementioned studies used appropriately conservative datasets to search for TE-derived protein coding sequences, it may also be the case that the primary detection methods they employed are insufficiently sensitive since they rely on DNA-DNA sequence comparisons. For instance, RepeatMasker, which is the most widely used program for the detection of TE sequences, uses pairwise comparisons of genomic DNA sequences with DNA consensus sequences that represent TE families. Protein sequence based similarity searches are more sensitive than DNA based searches, and profile searches that take advantage of information on site-specific variation along protein domains are proven to be the most sensitive approach for detecting sequence homology [18-20].

The increased sensitivity of protein and profile based searches is underscored by two recent studies that uncovered many more putative cases of TE-derived protein coding sequences. Roy Britten compared human protein coding sequences to the Repbase library of consensus TE sequences [21,22] using both RepeatMasker and a protein sequence based approach that used six-frame translations of Repbase sequences. Use of the protein (translated) sequence search method resulted in a more than two-fold increase, from 814 to 1,950, in the number of genes found to have TE-derived protein coding sequences [23]. An even more sensitive profile based search method was used by Zdobnov *et al*. to search for TE-derived protein coding sequences in four vertebrate genomes [24]. These authors compiled a set of known protein domains that are characteristic of TEs, and profiles of these domains were then used in hidden Markov model (HMM) searches of the protein sequences. This analysis resulted in the discovery of 1,000 vertebrate genes containing protein coding sequences that are related to TEs. However, neither the Britten nor the Zdobnov *et al*. studies confined their searches to experimentally characterized protein coding sequences as did the studies of Pavlicek *et al*. and Gotea and Makalowski, both of which resulted in far smaller estimates for the fraction of genes with TE-derived protein coding sequences.

Clearly, the extent to which TEs contribute protein coding sequences to vertebrate genomes is not a settled matter. Relatively insensitive searches of conservative data sets lead to low estimates for the fraction of TE-derived protein coding sequences, while more sensitive searches of less conservative data sets yield higher fractions. The aims of this study are i-to evaluate the ascertainment biases related to different sequence similarity search methods and ii-to try and better understand the potential of TEs to contribute protein coding sequences to vertebrate genomes. To these ends, we searched conservative, experimentally characterized, protein coding sequence data sets for TE-derived sequences using sensitive profile based search methods. We also compared the results of profile based search methods with more traditional pairwise DNA and protein based search methods. Known cases of experimentally characterized proteins with TE-related sequences were used as positive controls to assess the sensitivity of the different sequence similarity search techniques. Finally, we used probabilistic gene prediction methods as well as an analysis of relative nucleotide (GC) frequencies across codon positions to evaluate the protein coding probability of TE-derived exon sequences.

## Results and Discussion

### Searching for TE-associated proteins

We used a number of approaches to detect molecular domestication events, specifically exaptation of host (cellular) CDS from TE sequences, by searching for the presence of TE-related sequences in functionally well characterized host protein sequences and CDS. A total of 41,492 PDB entries and 21,050 Swiss-Prot directly sequenced proteins were taken to represent functionally well characterized proteins (genes) since they have been experimentally determined. Viral proteins were excluded from these data sets in order to avoid the overlap among protein domains shared between viral and retrotransposon-encoded proteins resulting in final data sets of 39,252 PDB and 20,732 Swiss-Prot entries. Using the combined automatic and manual search procedure described in the Methods section, we identified 124 TE-related Pfam protein domains (See Additional file 1). We then searched for the presence of these TE-related domains among the experimentally characterized PDB and Swiss-Prot data sets using profile-based similarity search methods (HMM profiles) as described in the Methods section. The numbers (percentages) of protein sequences found to possess TE-related domains, based on a series of increasingly stringent HMM search cut-off criteria, are shown in Table 1 and Table 2 for the PDB and Swiss-Prot data sets respectively.

**Table 1:** Detection of TE-encoded sequences in PDB proteins. The number of PDB entries found with TE protein fragments (from autonomous TEs) by different search programs is shown. The percentage of total PDB entries is shown in the parenthesis. The square bracket indicates the number and the percentage of protein entries associated with sequences derived from TEs including the non-autonomous ones.

Cut-off value	HMMER	BLASTN	BLASTP	BLASTX	TBLASTN	TBLASTX	RM
E-value ≤ 1	17543 (44.69%)	4924 (16.15%) [10890: 35.72%]	1558 (3.97%)	1643 (4.19%) [3343: 8.52%]	8662 (28.41%)	13107 (42.99%) [19891: 65.25%]	N/A
E-value ≤ 0.1	2757 (7.02%)	1531 (5.02%) [3076: 10.09%]	614 (1.56%)	764 (1.95%) [1207: 3.08%]	5671 (18.60%)	7721 (25.33%) [11474: 37.64%]	N/A
E-value ≤ 0.01	533 (1.36%)	778 (2.55%) [1453: 4.77%]	424 (1.08%)	586 (1.49%) [827: 2.11%]	3943 (12.93%)	5688 (18.66%) [8481: 27.82%]	N/A
E-value ≤ 0.001	256 (0.65%)	564 (1.85%) [917: 3.01%]	364 (0.93%)	530 (1.35%) [700: 1.78%]	3030 (9.94%)	4832 (15.85%) [6995: 22.94%]	N/A
E-value ≤ 0.0001	168 (0.43%)	423 (1.39%) [682: 2.24%]	308 (0.78%)	464 (1.18%) [552: 1.41%]	2266 (7.43%)	4057 (13.31%) [6033: 19.79%]	N/A
E-value ≤ 0.00001	148 (0.38%)	371 (1.22%) [555: 1.82%]	210 (0.54%)	388 (0.99%) [474: 1.21%]	1676 (5.50%)	3533 (11.59%) [5035: 16.52%]	N/A
GA (gathering threshold)	140 (0.36%)	N/A	N/A	N/A	N/A	N/A	N/A
TC (trusted cut offs)	140 (0.36%)	N/A	N/A	N/A	N/A	N/A	N/A
default value	N/A	N/A	N/A	N/A	N/A	N/A	465 (1.53%) [950: 3.12%]

**Table 2:** Detection of TE-encoded sequences in SwissProt directly sequenced proteins. The number of SwissProt directly sequenced proteins found with TE protein fragments (from autonomous TEs) by different search programs is shown. The percentage of total SwissProt entries is shown in the parenthesis. The square bracket indicates the number and the percentage of protein entries associated with sequences derived from TEs including the non-autonomous ones.

Cut-off value	HMMER	BLASTN	BLASTP	BLASTX	TBLASTN	TBLASTX	RM
E-value ≤ 1	8182 (39.47%)	2108 (16.39%) [4468: 34.74%]	2909 (14.03%)	3030 (14.62%) [4620: 22.28%]	3418 (26.58%)	5052 (39.28%) [7576: 58.91%]	N/A
E-value ≤ 0.1	1368 (6.60%)	655 (5.09%) [1331: 10.35%]	1481 (7.14%)	1632 (7.87%) [2185: 10.54%]	2159 (16.79%)	3009 (23.40%) [4501: 35.00%]	N/A
E-value ≤ 0.01	214 (1.03%)	316 (2.46%) [573: 4.46%]	935 (4.51%)	1103 (5.32%) [1503: 7.25%]	1559 (12.12%)	2180 (16.95%) [3208: 24.95%]	N/A
E-value ≤ 0.001	65 (0.31%)	187 (1.45%) [351: 2.73%]	694 (3.35%)	844 (4.07%) [1186: 5.72%]	1204 (9.36%)	1863 (14.49%) [2668: 20.75%]	N/A
E-value ≤ 0.0001	30 (0.14%)	112 (0.87%) [235: 1.83%]	516 (2.49%)	668 (3.22%) [971: 4.68%]	882 (6.86%)	1607 (12.50%) [2236: 17.39%]	N/A
E-value ≤ 0.00001	19 (0.09%)	83 (0.65%) [181: 1.41%]	372 (1.79%)	516 (2.49%) [776: 3.74%]	653 (5.08%)	1403 (10.91%) [1926: 14.98%]	N/A
GA (gathering threshold)	14 (0.07%)	N/A	N/A	N/A	N/A	N/A	N/A
TC (trusted cut offs)	14 (0.07%)	N/A	N/A	N/A	N/A	N/A	N/A
default value	N/A	N/A	N/A	N/A	N/A	N/A	154 (1.20%) [336: 2.61%]

To compare the sensitivity of the HMM profile-based search method with more standard sequence-against-sequence similarity search methods, we used the BLAST and RepeatMasker programs to search for TE-derived sequences among host proteins and their corresponding CDS. To this end, we built CDS databases corresponding to the PDB and Swiss-Prot protein data sets, which contain 34,795 and 38,754 CDS sequences, respectively. These CDS data sets correspond to 30,486 PDB and 12,860 Swiss-Prot proteins. The difference in the number of proteins versus CDS can be attributed to the fact that a number of protein sequences lack the matching CDS because they are synthetic, mutated, or chimeric proteins. In addition, some protein entries may be related to more than one CDS sequence, while some CDS may match with several PDB entries due to the redundancy of protein chains. For use as query sequences in BLAST searches, we created three TE sequence libraries from data provided in Repbase: 5,611 TE sequences (for all TEs in all taxa), 1,423 TE-encoded proteins and 1,349 TE CDS sequences. The specific combinations of BLAST program, query set and data base set used in each search is shown in Table 3. The numbers (percentages) of sequences found with TE-related domains, based on a series of increasingly stringent E-value cut-offs, are shown in Table 1 and Table 2 for the PDB and Swiss-Prot data sets respectively. Finally, the RepeatMasker program was used to search for TE-related sequences among the PDB and Swiss-Prot CDS data sets (see numbers and percentages of hits in Table 1 and Table 2).

**Table 3:** Sequence similarity program-query-database combinations used to search for TE-related host sequences

Tool	Query	Database
HMMER	PDB/Swiss-Prot protein	HMM profiles of TE-related Pfam domains
BLASTN	TE CDS & all TE sequences	PDB/Swiss-Prot CDS
BLASTP	TE protein	PDB/Swiss-Prot protein
BLASTX	TE CDS & all TE sequence	PDB/Swiss-Prot protein
TBLASTN	TE protein	PDB/Swiss-Prot CDS
TBLASTX	TE CDS & all TE sequence	PDB/Swiss-Prot CDS
RepeatMasker	PDB/Swiss-Prot CDS	TE CDS & all TE sequences

Considering the results of the three different classes of search strategies – HMMER, BLAST and RepeatMasker – together yields some unexpected results. Not surprisingly, however, RepeatMasker proved to be the least sensitive strategy to search for TE-related host protein coding sequences. Using the fairly liberal default cut-off value, which returns a number of hits with marginal reliability, RepeatMasker yields a lower number of hits than all but the most conservative searches with the other methods (Table 1 and Table 2). This is consistent with the fact that RepeatMasker relies on DNA-DNA sequence comparison.

**Figure 1:**
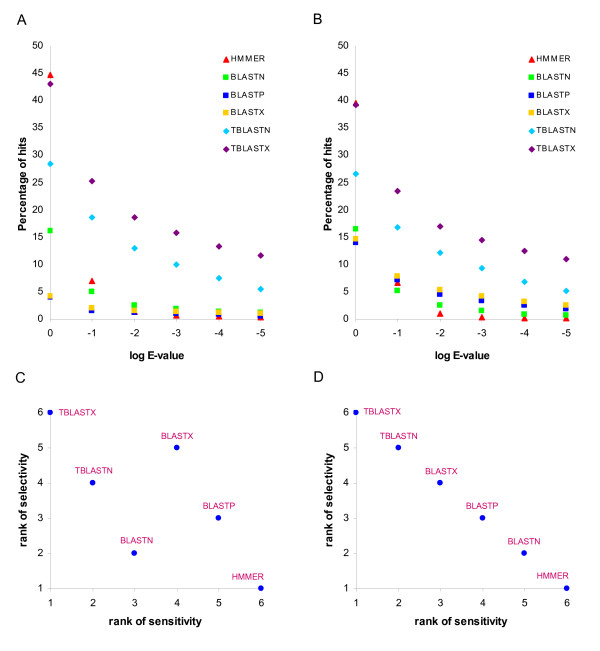
**Sensitivity and selectivity comparison for different sequence similarity search methods**. The percentage of hits returned by different sequence similarity search methods are shown across increasingly stringent E-value cut-offs for the PDB (A) and SwissProt (B) data sets. The selectivity and sensitivity ranks are compared for different search methods for the PDB (C) and SwissProt (D) data sets.

To compare the results of the HMMER versus BLAST search strategies, we plotted the percentage of hits against the E-value threshold used (Figure 1A and 1B). Together with Tables 1 and 2, these plots show the relative numbers (percentages) of hits retrieved using each method. TBLASTX searches, where CDS are translated in all six reading frames and are searched against nucleotide databases that are translated in six frames, gave the highest number of hits across all but the most liberal E-value cut-offs. This is consistent with previous results, showing that translated BLAST searches yield far more TE-host protein similarity than BLASTN or RepeatMasker searches [23]. The profile-based HMMER searches, which are expected to be the most sensitive, did return the highest number of hits at liberal E-values, but after two rounds of decreasing E-values, HMMER dropped off to yield the fewest number of hits across all the methods (Table 1, Table 2 and Figure 1). Thus, HMMER appears to be particularly sensitive to increasingly stringent E-value cut-offs.

To evaluate the selectivity of the search methods we employed, we measured the exponential rate of decline in the relative number (percentage) of hits retrieved at decreasing E-value thresholds, which allowed us to measure the effect of increasing stringency on the number of hits retrieved across methods. This was done by fitting exponential trend lines to the data shown in Figure 1A and Figure 1B and then ranking the searches with respect to the exponent of the trend line; the most selective methods are ranked the highest (*i.e*. have the lowest rank number). In this way, HMMER was shown to be the most selective method and TBLASTX the least selective. As could be expected, selectively is inversely correlated with sensitivity, and exactly so for the SwissProt search, as can be seen when the ranks of method sensitivity (number of hits) are compared to the selectivity ranks (Figure 1C and Figure 1D). Again, this overall trend defied the expectations of increased sensitivity of profile methods that we had at the outset of the study.

**Figure 2:**
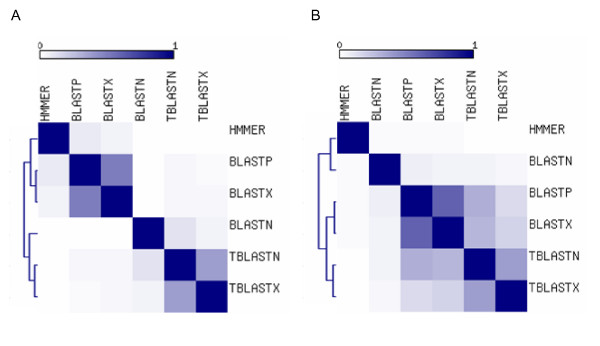
**Relationships among sequence similarity search methods**. Colors represent the fraction hits shared between methods, from 0 (white) to 1 (purple). The matrices are symmetrical with self-similarity shown along the diagonal. The search methods are ordered along both axes of the plots with respect to similarity, and dendograms showing the relationships among methods are shown for the PDB (A) and SwissProt (B) data sets.

We also considered the relationships among the different search methods in terms of the fraction of hits that they had in common. For each pair of search methods, the fraction of shared hits was calculated (see Methods), and the resulting pairwise similarity matrix was used to cluster the methods (Figure 2). For both the PDB and SwissProt searches, the translated BLAST methods group together as do the protein searches BLASTP and BLASTX. BLASTN was more similar to the translated methods in the PDB search, while it had lower overlap with the other BLAST methods in the SwissProt search. HMMER consistently showed the lowest overlap with other methods. Perhaps more importantly, the extent of overlap between the different methods was surprisingly low. For instance, at the lowest E-value cut-off only 2 out of a total of 4,241 hits for PDB and 2 out of 1,724 for SwissProt were identified by all six search methods. This underscores the fact that the different search methods are very much complementary and indicates that an exhaustive search for potential TE-CDS exaptation events will require the use of a variety of search techniques.

### Comparative analysis of cases of TE-CDS exaptation

HMMER was also run using the most conservative gathering (GA) and trusted cut-off (TC) thresholds described in the Methods section. Searches using GA and TC yield the fewest number of hits for both the PDB and Swiss-Prot searches. Thus, we took these results to be the most reliable (conservative) set of TE-related host proteins and further evaluated these results to look for *bona fide *cases of TE-CDS exaptation.

By manually evaluating these results, we were able to classify the hits into five distinct categories (see Methods), only one of which represents the kinds of TE-CDS exaptation events that we are most interested in (Table 4). For instance, the vast majority of apparent TE-related proteins in the PDB data set corresponded to either synthetic constructs (*i.e*. artificial sequences) or non-specific, and often ubiquitous, TE-related protein domains such as RNaseH. For this latter category, the non-specific TE-related domains, it is a formal possibility that they represent ancient TE-CDS exaptation events but it is difficult, if not impossible, to unambiguously support that assertion. Other proteins detected in the PDB set correspond to TE-encoded proteins and viral proteins. Only 11 out of 140 cases (or 7.9%) correspond to likely TE-CDS exaptation events. With the GA and TC thresholds, the Swiss-Prot dataset yielded far fewer total hits than did PDB and only 3 of these correspond to likely TE-CDS exaptation events (Table 4).

**Table 4:** Classification of proteins containing TE-associated Pfam domains detected by the GA and TC cut-offs of HMMER. The categories of hits are described in the text and the number (percentage) for each category is shown for searches against the PDB and SwissProt data sets.

Category	PDB	Swiss-Prot
Potential TE-related proteins	11 (7.86%)	3 (21.43%)
Viral proteins	14 (10.00%)	0 (0%)
TE-encoded proteins	18 (12.86%)	7 (50.00%)
Synthetic construct	47 (33.57%)	0 (0%)
non-specific TE-related protein domains	50 (35.71%)	4 (28.57%)

A set of 12 likely TE-CDS exaptation events, representing the non-redundant union of the most reliable cases from the PDB and Swiss-Prot sets in Table 4, were further analyzed in order to assess the ability of BLAST and RepeatMasker to detect these cases. Only one of the 12 proteins was detected using all methods, and again, RepeatMasker was shown to be the least sensitive method (Table 5). Indeed, as expected, DNA-DNA search methods in general were found to be insensitive; there are 4 cases where BLASTN and RepeatMasker are the only programs unable to detect the TE-CDS similarity. There were four individual cases, corresponding to two different Pfam domains, where only HMMER was able to detect the TE-protein sequence similarity. These results stand in contrast to the results of the previous section, which indicate that HMMER is the least sensitive search method overall. There are two possible explanations for this dissonance. First of all, HMMER may suffer from a lack of coverage due to its reliance on the collection of Pfam domain family definitions. Secondly, and perhaps more plausible, the different search methods may in fact be complementary in terms of detecting different sets of exaptation events. This may be particularly relevant for DNA based, and/or translated, search methods that are able to compare non-coding TE-derived sequences to host protein and CDS sequences.

**Table 5:** Analysis of the qualified set of TE-associated domain containing proteins. Twelve PDB/Swiss-Prot proteins with TE-associated Pfam domains detected by HMMER (GA and TC cut-offs) are shown. The results from BLAST and RepeatMasker analysis are compared (√ = found, X = not found TE-related sequence). The cut-off E-value of 0.01 was used as the detection criteria.

Accession	Name	Organism	Pfam domain	BLASTN	BLASTP	BLASTX	TBLASTN	TBLASTX	RM
2jm3	Hypothetical protein	*C. elegans*	THAP	X	X	X	X	X	X
1a0p, XERD_ECOLI	Tyrosine recombinase xerD	*E. coli*	Phage_integrase	X	X	X	X	X	X
1bw6, 1hlv	Centromere protein B	*H. sapiens*	CENP-B_N	√	√	√	√	√	X
1uhu	retroviral Gag MA-like domain of RIKEN cDNA 3110009E22	*M. musculus*	Gag_MA	N/A	√	N/A	√	N/A	N/A
1y4m	Syncytin-2	*H. sapiens*	TLV_coat	√	√	√	√	√	√
2a3v	Site-specific recombinase IntI4	*V.cholerae*	Phage_integrase	X	X	X	X	X	X
2cqf	Lin-28 homolog A (Zinc finger CCHC domain-containing protein 1)	*H. sapiens*	zf-CCHC	X	√	√	√	√	X
2ct5	Zinc finger BED domain-containing protein 1	*H. sapiens*	zf-BED	X	√	√	√	√	X
2d8r	THAP domain-containing protein 2	*H. sapiens*	THAP	X	√	√	√	√	X
2djr	Zinc finger BED domain-containing protein 2	*H. sapiens*	zf-BED	X	√	√	√	√	X
CBH1_SCHPO	CENP-B homolog protein 1	*S. pombe*	DDE	X	√	√	√	√	X
XERC_ECOLI	Tyrosine recombinase xerC	*E. coli*	Phage_integrase	X	X	X	X	X	X

### Case studies of known TE-derived genes

There are a number of well verified cases of host proteins (genes) that are known to have been derived from TE sequences. These are proteins that have been shown to be functionally analogous and evolutionarily derived from their TE-encoded counterparts. For instance, the enzyme Telomerase evolved from TE-encoded reverse transcriptase enzymes [25,26] and the RAG1 recombinase is related to the transposase enzymes [27,28]. The centromere protein CENPB [29] and SETMAR [30] are other well documented cases of the evolution of host CDS from TEs. We have used these cases as positive controls in order to further evaluate the ability of the different classes of search methods to detect cases of TE-CDS exaptation.

We assessed the ability of each program to detect human proteins or CDS for all four of these cases (Table 6). Translated BLAST searches BLASTX and TBLASTN were the most sensitive search methods finding all of the cases in this data set, and HMMER was shown to be fairly sensitive in detecting three out four of the known cases of TE-exaptation. RepeatMasker was the least sensitive detecting only the SETMAR case. SETMAR represents an evolutionarily recent TE-CDS exaptation event that occurred during the primate radiation some 40–58 million years ago [30]. Thus, the SETMAR CDS retains DNA sequence similarity to the Hsmar1-type TE transposase gene from which it is derived. In any case, all the search methods were able to detect SETMAR, so RepeatMasker would not be necessary to elucidate this case. In general, for the BLAST searches, translated and protein based searches are the most sensitive followed by DNA-based BLASTN.

**Table 6:** Detection of previously identified TE-associated proteins. The ability of the different sequence similarity search methods to detect well known cases of TE-derived CDS is indicated with √ and failure to detect is indicated with X.

Name	TE-protein	HMMER	BLASTN	BLASTP	BLASTX	TBLASTN	TBLASTX	RM
Telomerase	Reverse transcriptase (LINEs)	√	X	X	√	√	√	X
RAG1	Transposase (Transib superfamily)	X	X	√	√	√	X	X
CENPB	pogo-like DNA transposase	√	√	√	√	√	√	X
SETMAR	Hsmar1 transposase	√	√	√	√	√	√	√

### Evolutionary relationship between TE and cellular proteins

In the formal sense, establishing a solid, statistically significant, sequence similarity relationship between TE-encoded and cellular proteins is necessary but not sufficient to make the claim of a TE-CDS exaptation event. This is exemplified by the numerous cases of ubiquitous, non-specific TE-related protein domains uncovered when searching the PDB and Swiss-Prot experimentally characterized data sets (Table 4). These abundant protein domains, such as RNaseH, can be functional analogs that have evolved convergently in host and TE genomes or they may have their evolutionary origins in host (cellular) genomes and been subsequently captured by TEs. Thus, it is necessary to document the evolutionary relationships between TE-encoded and related host-encoded protein domains as accurately as possible in order to evaluate the evidence for TE-CDS exaptation. Phylogenetic analysis is best suited to this task. Indeed, phylogenetic analysis is needed to unequivocally demonstrate a TE-origin, *i.e*. the direction of the TE-to-host sequence transfer, for protein domains with similarity between TEs and host genomes as was shown for the case of Telomerase [26].

To illustrate this analytical process, we have chosen the THAP protein domain. Sequence similarity between the THAP domain and TEs has been noted previously but the evolutionary origins of the domain, and in particular the specific direction of the TE-host transfer, remains uncertain. The *Caenorhabditis elegans *C-terminal binding protein (CtBP) [PDB: 2jm3] contains the THAP domain, a ~90 residue domain, which is restricted to animals and shared between the THAP family of cellular DNA-binding proteins and transposases encoded by DNA-type TEs. This domain was previously found to be homologous to the site-specific DNA-binding domain (DBD) of *Drosophila *P-element transposase [31]. An evolutionary analysis of the domain architectures and sequence similarities among THAP domain containing proteins was taken to suggest the possibility that cellular proteins have recruited this domain on more than one occasion [32].

In order to characterize all sequence relationships between TE and host-encoded THAP domains, we used HMMER with the Pfam THAP domain HMM profile to search among the Repbase library of TE-encoded proteins. The use of HMMER was necessitated by the fact that, consistent with results reported in previous sections of the manuscript, BLAST and RepeatMasker can not detect any TE-related sequence in *C. elegans *CtBP. Using HMMER, we found that PROTOP is the identity of the autonomous *Drosophila melanogaster *P element that contains the THAP domain, in positions 12 to 94 of its consensus protein sequence. We also identified six additional TE families containing THAP domain (KBOC_DB, P1_AG, P3_AG, P4_AG, Kolobok-1_XT, Kolobok-2_BF). In addition, CtBP was used as a BLASTP search query to identify host (cellular) genome encoded THAP domains. All TE and host encoded THAP domains were aligned, globally and locally, and phylogenetically analyzed as described in the Methods section.

**Figure 3:**
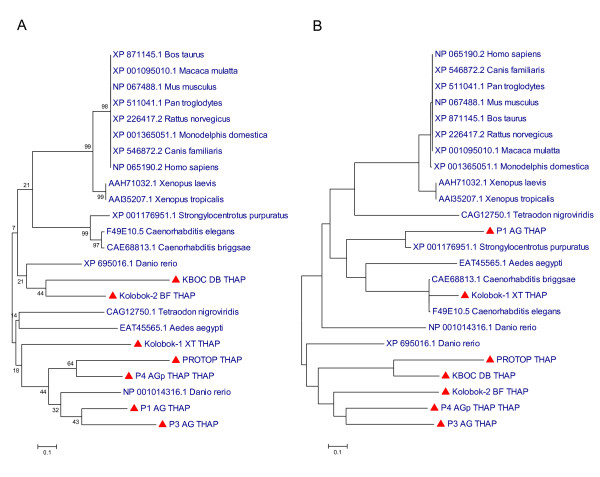
**Phylogenetic relationship of TE and cellular THAP domains**. Neighbor-joining trees of seven THAP homologous TE sequences and seventeen cellular THAP sequences from various species are shown. The trees were created based on (A) the multiple sequence alignment of all THAP sequences and (B) the pairwise gamma distance matrix calculated from BLAST all-against-all pairwise alignments. TE-THAP sequences are indicated by red triangle marks. Bootstrap values (A) represent the fraction of times that internal branches, supporting clades, were recovered among trees built from 1,000 re-sampled alignments.

The global and local alignment based phylogenetic analyses consistently identify one clade of host encoded THAP domains and a second clade of THAP domains encoded by both TEs and cellular genomes (Figure 3). Interestingly, the TE and host encoded domains are distributed throughout this clade suggesting the possibility of multiple transfers of THAP domain CDS between TE and host genomes. In addition, TE encoded THAP domains appear to have greater sequence diversity, reflected by the branch lengths, than host encoded THAP domains, consistent with a TE origin of the domain. Thus, it appears that THAP indeed evolved among TE sequences and was subsequently transferred on more than one occasion to host (cellular) genomes.

### Protein coding potential of TE-derived exons

By now, it is well known that TE-derived sequences are frequently incorporated into the exons of host mRNAs [12]. What is less clear is the extent to which TE-derived exons of host genes are destined to become protein coding sequences. Previously, we addressed this issue by searching functionally well characterized protein coding sequences for the presence of TE-related domains. Here, we take a DNA sequence codon based approach to this question. Our approach is based on the fact that protein coding sequences show a specific and marked periodicity of nucleotide frequencies across the first, second and third codon positions. This periodicity serves as a robust signal for a number of gene prediction algorithms, one of the earliest and most prominent example of which is GeneMark [33]. GeneMark can accurately identify protein coding nucleotide sequences based solely on the distribution of observed nucleotide frequencies across codon positions.

**Figure 4:**
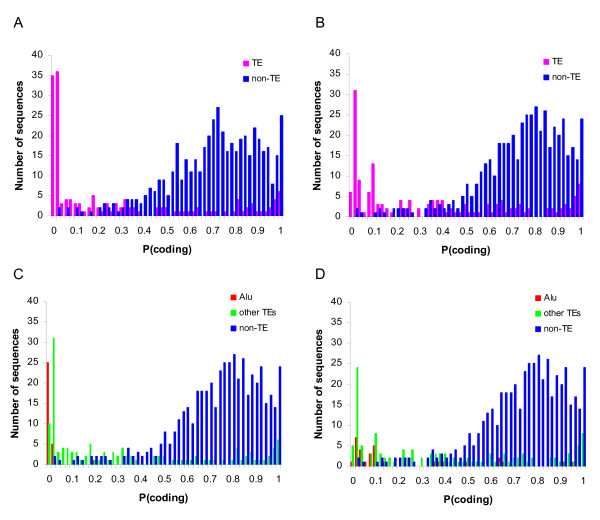
**Coding probability of human CCDS genes**. The coding probability of TE-derived coding sequences (pink) and non TE-derived coding sequences (blue) are shown, with results from the original GeneMark model (A) and our custom trained GeneMark model (B). TEs are separated in Alu (red) and non-Alu (green) for the original (C) and custom (D) GeneMark models.

We used the eukaryotic version of GeneMark [33], to evaluate the coding capacity of TE-derived exon sequences in the human genome. First, we compared the locations of 14,802 consensus CDS (CCDS) genes mapped to the hg17 build, from the UCSC Genome Browser [34], of the human genome to the locations of annotated TEs (see Methods). 761 of the human CCDS genes have TE-derived exon sequences; there are a total of 817 TE-derived exons. The 761 human genes with TE-derived exons include 160 TE-derived fragments with the minimum length of 100 nt required for GeneMark analysis. Using GeneMark probabilistic models (see Methods), we analyzed the TE-derived exon sequences as well as 500 randomly chosen representative non TE-derived exons by calculating their probability to be protein coding regions. The distributions of protein coding potentials (probabilities) for TE versus non TE sequences are shown in Figure 4. Visually the distributions are quite distinct, with TE derived exons having far lower coding potential, and accordingly there is a highly significant difference between the two coding probability distributions, *D *= 0.67 *P *= 0 Kolmogorov-Smirnov test (Figure 4A). The average coding potential of TE-derived exons was 0.26 compared to 0.70 for non TE-derived coding sequences. Using a more sensitive custom-trained GeneMark model gave consistent results, 0.35 average TE coding probability versus 0.73 for non TE sequences with significantly different distributions *D *= 0.59 *P *= 0 Kolmogorov-Smirnov test (Figure 4B). Clearly, TE-derived exons have much lower coding probability than non-TE derived sequences suggesting that many of these exons do not actually encode proteins.

Since the TE-derived exons evaluated using GeneMark as described above are taken from the RepeatMasker annotations on the human genome sequence, they do not include more ancient well established cases of TE-derived CDS such as the first three cases shown in Table 6. One would expect that these TE-derived CDS have higher protein coding potentials than the more recently exonized TE sequences revealed by RepeatMasker. In fact, when analyzed using GeneMark in the same way as described for the entire set of TE-derived exons, all of their protein coding probabilities are significantly greater (*z*-test: 15.5 <*z *< 16.9) than the average protein coding probability (0.35) of the aforementioned set of TE-derived exons: Telomerase = 0.81, RAG1 = 0.77, CENPB = 0.89. Interestingly, the protein coding probability of the relatively recent case of TE-CDS exaptation, SETMAR (0.67), is also significantly greater (*z *= 11.8) than the average coding potential for the set of RepeatMasker identified TE-derived exons. This is consistent with the fact that, while SETMAR does represent a recent case of TE-CDS exaptation, the particular TE-sequence that was exonized was already a protein-coding domain prior to becoming a host gene [30].

Taken together, these protein coding probability data are consistent with previous studies that have suggested caution is warranted when extrapolating genome sequence analyses to infer TE-CDS exaptation events [13,16,35,36]. In particular, the notion that non-autonomous TEs that do not encode any protein, including SINEs such as the Alu family of elements, can emerge as protein coding sequences after being incorporated into exons has been directly challenged [13]. On the other hand, Alus are frequently incorporated into mRNAs as exons [37-40], and there are a number of specific cases of Alu-derived CDS that have been proposed to provide novel CDS to primate genes [41,42]. In light of this controversy, we have specifically evaluated the potential coding capacity of Alu-derived exons using GeneMark.

Alu-derived exons were considered separately from all other TE-derived exons and their coding probability distributions were plotted along with the distribution for non TE-derived exons (Figure 4C and Figure 4D). Alu-derived exons have coding probability distributions that are shifted to the left, *i.e*. towards lower probability, than all other TE-derived exons. Indeed, the average coding probabilities for Alu-derived exons are significantly lower than the averages for all other TE-derived exons (Table 7). This result holds under a number of different analytical conditions (see Methods), including the two different GeneMark models and the consideration of Alu-derived exons as only containing Alu sequences or containing Alu plus other TE sequences (composite TE-exons in Table 7).

**Table 7:** Comparison of protein coding potential for Alu-derived exons versus other TE-derived exons. Average protein coding potentials are compared between the specific pairs of groups indicated using the Student's t-test. Comparisons were done using two GeneMark models: pre-trained and custom-trained (see Methods).

GeneMark model	Comparison groups	mean	df	*t*	*P*
Pre-trained	Alu-exons vs other TE-exons	0.0069 vs 0.3229	158	9.8	5.2e-18
	Alu-containing composite TE-exons vs other TE-exons	0.0135 vs 0.3417	158	9.6	1.3e-17
Custom-trained	Alu-exons vs other TE-exons	0.2034 vs 0.3802	158	2.9	4.3e-3
	Alu-containing composite TE-exons vs other TE-exons	0.2033 vs 0.3920	158	3.4	7.4e-4

**Figure 5:**
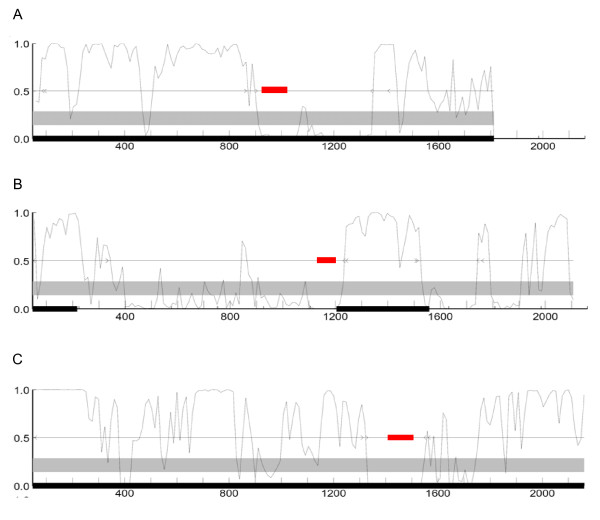
**Coding probability of genes with Alu-derived exons**. GeneMark protein coding probability analyses are shown for three genes with well-characterized Alu-derived exons: C-rel-2 [CCDS: CCDS1864.1] (A) MTO1-3 [CCDS: CCDS4979.1] (B) and PKP2b-4 [CCDS: CCDS8731.1] (C) [41]. Coding probabilities were calculated within windows sliding along the length of the genes. The locations of the Alu-derived exons are shown in red.

In addition to the global analysis of Alu-derived exon protein coding potential, we also evaluated several documented cases of Alu exonization events that are assumed to represent TE-CDS exaptations [41,42]. For these cases, the specific evolutionary scenarios giving rise to the Alu-derived exons are quite well documented, but the protein coding potential of the Alu-exons appears to be assumed. Here, the GeneMark web server [43], which runs both GeneMark and GeneMark.hmm [44] programs, was used to plot protein coding probabilities along the length of the CDS using a sliding window (Figure 5). This allowed the protein coding potential of the Alu-derived exons to be directly compared to that of the non TE-derived exons in the same genes. Consistent with their status as protein coding genes, the coding sequences analyzed tend to show uniformly high protein coding probabilities. However, the Alu-derived exons show far lower protein coding potential than the rest of the gene sequences. The apparent low coding potential of Alu derived exons may also reflect the fact that these sequences have a relatively recent evolutionary origin as exons and thus have not had enough time to accumulate the kinds of changes that would yield periodicities that more closely resemble other coding sequences.

### GC codon distribution for TE-derived exons

The distribution of GC content across codon positions can also be used to evaluate the protein coding potential of genomic sequences. This kind of analysis is based on the fact that the GC level (%G+C) is distinctly lower in the second (GC2) than in the third (GC3) codon positions for protein coding sequences in species ranging from human to *Escherichia coli *[45,46]. Thus, for protein coding sequences, regression analysis of %GC2 × %GC3 should yield a trend line with a slope y << 1. Here, we used GC2/GC3 regression analysis to compare the protein coding potential of TE-derived versus non TE-derived exons.

**Figure 6:**
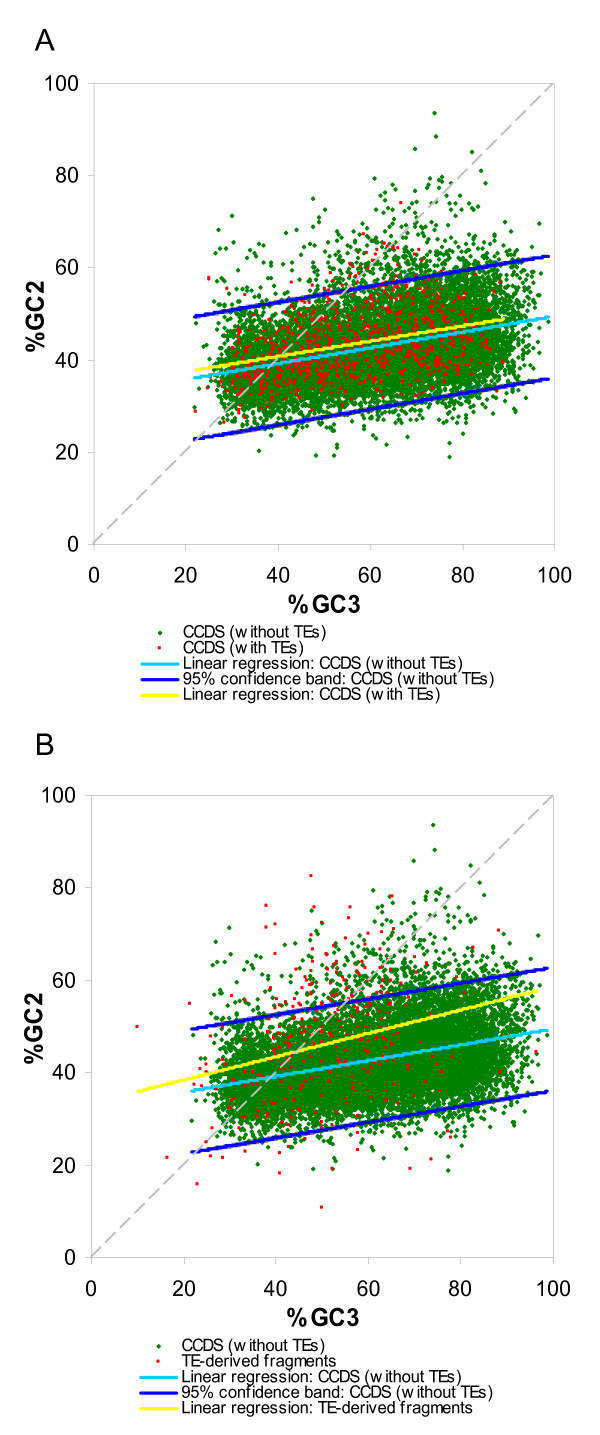
**The GC composition of human CCDS genes**. The scatter plots of %G+C of second (GC2) versus third (GC3) codon positions for TE-associated genes (red) and non-TE associated genes (green) are shown. The light blue line represents the linear regression line of non-TE associated genes while the blue lines show the 95% confidence interval. For the TE-associated group, the GC content for the whole sequence of TE-associated genes (A) and for TE-derived gene fragments only (B) are shown. The yellow line represents the linear regression line of these TE associated groups.

For the first analysis, GC2/GC3 trends were computed for entire genes that contain one or more TE-derived exons versus entire genes with no TE-derived exons (Figure 6A). In this case, the GC2/GC3 distributions are indistinguishable and do not have significantly different slopes (t = 0.36, df = 14,798, *P *= 0.71). However, 27.93% of TE associated genes were located outside the 95% confidence band of non-TE associated gene set. On the other hand, when TE-derived exons are considered alone (Figure 6B), the slopes of the TE-derived versus non TE-derived sets are significantly different (t = 2.84, df = 14,384, *P *= 4.6e-3), and 31.70% of TE-derived exons are found outside the 95% confidence interval for the non TE-derived set. Thus, while the GC2/GC3 analysis appears to suffer from a lack of resolution compared to the GeneMark coding potential analysis, it too points to a relatively low coding probability for TE-derived exons.

We also analyzed Alu-derived exons separately using GC2/GC3 codon analysis as was done with GeneMark. Visual inspection of the location of Alu-derived exons on the GC2/GC3 plot shows that they have relatively higher GC2, typical of non-coding sequence, and 41.79% fall outside the 95% confidence interval, all of which fall above the upper confidence interval boundary (See Additional file 2). In addition, Alu-derived exons have average GC2/GC3 ratios that are significantly higher than the GC2/GC3 ratios for all other TE-derived exons and for the non TE-derived gene set (Table 8). In other words, the GC2/GC3 analysis also suggests that Alu-derived exons are less likely to encode protein sequences than other TE-derived exons.

**Table 8:** Comparison of GC2/GC3 ratios for different classes of TE-derived and non TE genes (exons). Average GC2/GC3 ratios are compared for pairs of groups indicated using the Students' ttest.

Comparison groups	averages	df	*t*	*P*
TE-genes vs non TE-genes	0.82 vs 0.76	14800	7.4	1.2e-13
TE-exons vs non TE-genes	0.96 vs 0.76	14386	9.4	6.8e-21
Alu-exons vs other TE-exons	1.01 vs 0.95	345	1.9	6.2e-2
Alu-exons vs non TE-genes	1.01 vs 0.76	14106	8.2	3.9e-16

## Conclusion

The potential for TE sequences to become exapted as host protein coding sequences through the process of exonization has received a great deal of attention as of late [47-51]. Implicit in much of this literature is the assumption that exonized TE nucleotide sequences, *i.e*. TE sequences that are spliced into mRNAs, actually encode protein sequences. However, this assumption has been challenged on several different fronts [13,16,35,36]. In particular, it is unclear whether non-autonomous TEs that do not encode any protein, such as Alu elements, actually provide protein coding sequences after becoming exonized [13]. Nevertheless, recent studies continue to turn up numerous apparent cases of TE-CDS exaptation [23,24]. So the matter of TE-CDS exaptation remains unsettled, and in this report we have tried to address the issue from two perspectives: i-with respect to the ascertainment biases that arise from the use of different sequence similarity search methods and ii-in terms of the protein coding potential revealed by the probabilistic analysis of exonized TE nucleotide sequences.

Our use of profile-based (HMM) sequence similarity searches did allow for greater sensitivity than the more widely used DNA-DNA (*e.g*. RepeatMasker) search methods when employed on a test set of well-characterized exapted TE-CDS (Table 5 and Table 6). Thus, ascertainment biases could explain the paucity of reliable examples of TE-derived protein coding sequences uncovered via the analysis of experimentally characterized protein sequence data sets [13,16]. However, when profile-based search methods are similarly applied to large-scale datasets of experimentally characterized proteins, they did not turn up many more cases than previously found (Tables 1, Table 2 and Table 4). In fact, the profile-based search method appeared to be less sensitive than all BLAST-based search methods – nucleotide, protein or translated (Table 1 and Table 2). This apparent lack of power can actually be attributed to the superior selectivity of the profile-based methods (Figure 1) and suggests that many of the putative TE-CDS exaptation events turned up in BLAST searches may be spurious. In other words, profile-based search methods possess a valuable combination of sensitivity, measured by their ability to recover positive control test cases, and selectivity than any of the other search methods used. Nevertheless, the different search methods are complementary to the extent that combined search approaches are needed to thoroughly check any data set for all potential TE-CDS associations. Different search methods will also be more or less appropriate depending on the kind of exonization event that is being analyzed; for instance, it will not be possible to search for the contribution of non-coding TEs to exapted protein domains using profile methods based on protein sequence alignments.

The codon based analysis of exonized TE sequences suggests that many, if not most, of these sequences do not actually encode any protein. Non-coding TEs that are exonized, such as Alu, have particularly low protein coding probabilities. The lack of protein coding potential does not mean that exonized TE sequences are necessarily non-functional. They may in fact play a role in post-transcriptional gene regulation. We hypothesize that many exonized TE sequences serve as natural anti-sense transcripts, which can function as double stranded RNA regulators of gene (protein) expression. The repetitive dispersed nature of exonized TE sequences may provide a mechanism by which they can serve as master regulators with influence over the expression of numerous genes throughout the genome.

## Methods

### Detection of TE-encoded protein fragments

#### Sequence data sets

The set of functionally well characterized proteins was taken from two databases: Protein Data Bank (PDB) [14] (downloaded on 03/02/07) and Swiss-Prot Protein Database [15] (version 52.0). For the Swiss-Prot entries, only directly sequenced proteins were included in the data set. These directly sequenced proteins are the proteins whose amino acid sequence has been partially or completely determined experimentally by Edman degradation or by mass spectrometry and can be found by searching the Swiss-Prot database with the keyword 'Direct Protein Sequencing'. The data set of experimentally characterized protein sequences from PDB and Swiss-Prot was then filtered to remove the sequences from viruses. The nucleotide coding sequences (CDS) corresponding to the final set of protein sequences was obtained from EMBL CDS database [52]. It should be noted that PDB entries can contain more than one distinct protein sequence (chain) and the same protein sequence (chain) may be found in more than one PDB entry. A data set of protein sequences encoded by TEs and their corresponding CDS sequences were extracted from Repbase [21] version 12.02. The data set of all TE nucleotide sequences (including non-autonomous TEs) was retrieved from Repbase website [53].

#### Identification of TE-related protein domains

Protein domains that are associated with TEs were identified in version 21.0 of the Pfam database [20] and the associated InterPro annotation [54]. Pfam entries, both keywords and domain descriptions, were automatically searched using a set of related terms (e.g. transposon, retrotransposon, retroviral/retrovirus, transposase, reverse transcriptase, etc.) as in [24]. The resulting putative TE-related Pfam entries were then manually inspected to remove spurious hits corresponding to protein families that are not encoded by any TEs. Manual inspection was done using the Pfam domain descriptions and literature references. HMM profiles, representing the site-specific sequence variation, of the TE-related Pfam domains were used in searches with the HMMER program as described below.

#### Sequence similarity searches

The experimentally characterized PDB/Swiss-Prot protein sequence data sets described above were searched for the presence of the TE-related protein domains using version 2.3.2 of the HMMER program [55]. HMMER searches were run using a series of increasingly stringent threshold E-values, from E-value ≤ 1 to E-value ≤ 0.00001, in addition to the gathering threshold (GA) and trusted cut-off (TC) threshold values (Table 1 and Table 2). The GA and TC threshold cut-offs are values that have been bench-marked by the developers of HMMER to ensure that a minimum number of false-positive hits are detected. The GA thresholds are empirically set for each Pfam model and correspond to the score used to collect all of the sequences included in the Pfam full alignment. In other words, the GA threshold corresponds to the complete absence of false-positives. The TC threshold is similar to GA in the sense that it corresponds to the lowest scoring hit to any sequence included as a true member of a particular Pfam domain. TE-associated PDB/Swiss-Prot proteins detected by HMMER were classified into five categories: i-potential TE-related proteins (the host proteins containing TE-associated protein domains), ii-viral proteins (genuine viral proteins though the PDB source organism is not listed as a virus), iii-TE-encoded proteins found in TEs as opposed to cellular host proteins, iv-synthetic construct (synthesized protein sequences), and v-ubiquitous non-specific TE-related protein domains (*i.e*. host protein containing Pfam domains which are not specific to TE protein sequences but can be found in TEs as well).

Various BLAST programs [56] and the program RepeatMasker [17] were used to search the protein sequence and CDS data sets described above for TE-related protein sequences and/or TE-related CDS. The specific program-query-database combinations used for each search are shown in Table 3. BLAST programs were run using a series of E-value thresholds, from E-value ≤ 1 to E-value ≤ 0.00001, with default parameters and without low-complexity filtering.

The fraction (*f*) hits shared between any two methods was taken as the ratio of the number of hits retrieved in both searches to the total number of hits in both searches. For two searches that return *x *and *y *numbers of hits respectively:

fxy=x∩yx+y−x∩y
 MathType@MTEF@5@5@+=feaafiart1ev1aaatCvAUfKttLearuWrP9MDH5MBPbIqV92AaeXatLxBI9gBaebbnrfifHhDYfgasaacPC6xNi=xI8qiVKYPFjYdHaVhbbf9v8qqaqFr0xc9vqFj0dXdbba91qpepeI8k8fiI+fsY=rqGqVepae9pg0db9vqaiVgFr0xfr=xfr=xc9adbaqaaeGacaGaaiaabeqaaeqabiWaaaGcbaGaemOzayMaemiEaGNaemyEaKNaeyypa0tcfa4aaSaaaeaacqWG4baEcqWIPisscqWG5bqEaeaacqWG4baEcqGHRaWkcqWG5bqEcqGHsislcqWG4baEcqWIPisscqWG5bqEaaaaaa@3F03@

All pairwise similarity values were calculated in this way, and the resulting matrix was clustered using hierarchical clustering. Matrix clustering and visualization were done using the programs Genesis [57] and Matrix2png [58] respectively.

### Analysis of known cases of TE-derived proteins (genes)

Several well known cases of proteins (genes) derived from TEs were evaluated by the HMMER, BLAST and RepeatMasker programs to determine the efficiency of different search methods in detecting TE-CDS exaptation events. The TE sequence data set sources as described in the previous section were used for these searches. The Genbank sequence accessions for the known cases are Telomerase Reverse Transcriptase [RefSeq: NM_198253, NM_198255, NP_937983, NP_937986], Recombination Activating Gene 1 (*RAG1*) [RefSeq: NM_000448, NP_000439], Centromere protein B (*CENPB*) [RefSeq: NM_001810, NP_001801], SET domain and Mariner transposase fusion gene (*SETMAR*) [RefSeq: NM_006515, NP_006506].

### Evolutionary analysis of TE-associated protein domain

We used the THAP domain-containing protein, *C. elegans *C-terminal binding protein (CtBP) [PDB: 2jm3], for a phylogenetic analysis of THAP domain shared between TE and cellular proteins. The position of the THAP domain in *C. elegans *CtBP [RefSeq: NP_508983] was identified using HMMER program. The BLASTP program was used to search for the homologous sequences of CtBP THAP in other species, using the Genbank non-redundant database, and the sequence fragments corresponding to the THAP domain were extracted as "cellular THAP". The library of TE proteins sequences (described in the sub-section Detection of TE-encoded protein fragments: Sequence data sets) was searched for the THAP-containing entries by using HMMER program with gathering (GA) threshold cut-off. The sequence fragments corresponding to the THAP domain in TE proteins were extracted as "TE-THAP" sequences.

Phylogenetic analysis of THAP sequences was done using the neighbor-joining algorithm [59] implemented in the MEGA program [60]. Two sources of pairwise distances were used based on i-global sequence alignment of THAP domains with CLUSTALW [61] and ii-local alignment of THAP domains using all-against-all pairwise BLASTP. For the global THAP domain sequence alignments, Poisson distances were used, and for the local THAP domain comparisons, p-values (proportion of differences) taken from the BLAST output were transformed into gamma distances using α = 2.25 [62]. Bootstrap analysis, based on 1,000 replicates, was performed on the global THAP sequence alignment.

### Codon based analysis of TE-derived exons

The UCSC Genome Brower [34] and Table Browser tools [63] were used to search for human protein coding sequences co-located with TEs. Genomic locations of the CCDS genes mapped to the hg17 (NCBI Build 35) version of the human genome sequence were compared to the locations of TEs annotated with the RepeatMasker program [17]. The CCDS gene data set [64] was chosen because it represents a highly reliable set of gene models that are built from multiple lines of evidence and undergo quality analysis across several genomic centers before being released. Two data sets were created in this way: i-genes containing TE-derived exon sequences and ii-genes without TE-derived exons.

Version 2.5f of the GeneMark program [33] was used to compare the protein coding probabilities of TE-derived and non TE-derived human exons. GeneMark uses three-periodic inhomogeneous Markov models to analyze protein coding sequences and we used two models in our analysis. The first model is the GeneMark model pre-trained on validated coding and non-coding sequences of the human genome. This model is made available with the program. We also trained a customized GeneMark model using protein coding exon sequences from the non TE-derived gene set for the coding training set and intron sequences from the same genes as the non-coding training set. Each training set was classified into five groups based on %GC content (<41, 41–47, 47–53, 53–59, >= 59) for separate training of the fifth order Markov chain models. Note that 100 non TE-derived genes of each GC level were randomly selected as a set of non TE test sequences and removed from the training set before model training. The GeneMark program was run on the set of genes with TE-derived exons using the custom made model parameters corresponding to the GC content of each gene. The sliding window size was chosen to be 96 nt long and the step size to be 3 nt. The average posterior probability, which characterizes the probability that the sequence encodes a protein, was calculated for each TE-derived exon sequence fragments (>100 nt) using the following formula:

P(codx..y) =1n∑iP(cod1|F)
 MathType@MTEF@5@5@+=feaafiart1ev1aqatCvAUfKttLearuWrP9MDH5MBPbIqV92AaeXatLxBI9gBaebbnrfifHhDYfgasaacPC6xNi=xI8qiVKYPFjYdHaVhbbf9v8qqaqFr0xc9vqFj0dXdbba91qpepeI8k8fiI+fsY=rqGqVepae9pg0db9vqaiVgFr0xfr=xfr=xc9adbaqaaeGacaGaaiaabeqaaeqabiWaaaGcbaGaeeiuaaLaeeikaGIaee4yamMaee4Ba8Maeeizaq2aaSbaaSqaaiabbIha4jabb6caUiabb6caUiabbMha5bqabaGccqqGPaqkcqqGGaaiiiaacqWF9aqpjuaGdaWcaaqaaiabigdaXaqaaiabd6gaUbaakmaaqafabaGaeeiuaaLaeeikaGIaee4yamMaee4Ba8Maeeizaq2aaSbaaSqaaiabbgdaXaqabaGccqqG8baFcqqGgbGrcqqGPaqkaSqaaiabdMgaPbqab0GaeyyeIuoaaaa@4ABA@

where *x*+*W*/2 <= *i *<= *y*-*W*/2, *x *= start position of TE fragment, *y *= end position of TE fragment, *n *= # of sliding windows for which the midpoint lies within the range of *x*+*W*/2 to *y*-*W*/2, *i *= the midpoint of each sliding window, P(cod_1_|F) = posterior probability of the event that given the fragment F, it carries genetic code in frame 1 (starting from the very first nucleotide), *W *= the width of sliding window. The coding probability was calculated in the same way for the non TE test sequences. The analysis was repeated for the same test set using the pre-trained GeneMark models for human genome.

For the GC2/GC3 analysis, the GC level (%G+C) of second (GC2) and third (GC3) codon positions were calculated for each coding sequence of both the TE-derived and non TE-derived gene data sets. In addition, %GC2 and %GC3 were calculated for TE-derived fragments that are at least 60 nt long.

## Competing interests

The author(s) declare that they have no competing interests.

## Authors' contributions

JP participated in the design of the study, all of the analyses and the drafting of the manuscript. MTR and SP collected all TE-related Pfam domains and participated in the HMM searches. MB participated in the GeneMark analysis of protein coding sequences. IKJ participated in the design of the study and the drafting of the manuscript and coordinated all activities related to the study. All authors read and approved the final manuscript.

## Reviewers' comments

### Reviewer's report 1

Itai Yanai, Harvard University

In this manuscript Piriyapongsa et al. report a systematic analysis of the potential for transposable elements (TE) to contribute to proteomes. This is an issue of recent controversy in the literature with estimates of proteins with TE's in an organism ranging from 0.1% to 4%. The authors point out that along with the variation of estimates, is also variation of the datasets used and the searches invoked to analyze them. Datasets used comprise either predicted or experimentally-characterized protein sequences. Search methods used are DNA sequence similarity methods such as RepeatMasker, protein based searches, or profile based searches. The latter two methods are expected to be more sensitive than DNA searches. One specific goal of this study is to test whether the number of detected TEs is low in the clean set of experimentally-characterized protein sequences despite the use of sensitive sequence similarity measures. Another is to analyze the translation potential by two additional codon-based methods.

The authors find that even the more sensitive protein and profile methods do not uncover too many more instances of translated TE instances. This provides strong evidence that the original result – TEs exapted in 4% of the human proteome – is roughly off by an order of magnitude, when examined with respect to validated protein sequences as opposed to predictions of protein sequences. However, the most dramatic evidence for the general non-translation of TE's comes from Piriyapongsa et al.'s codon-based analysis which shows that they are as untranslated as ALUs. This dramatic lack of coding potential as estimated by the GeneMark program does however beg the question of why these have apparently maintained an open reading frame and did not accumulate non-sense mutations by drift.

One remaining open question now that the extent of TE exaptation is roughly estimated is the exact delineation of the TE proteins. One attractive method is the phylogenetic approach which Piriyapongsa et al. demonstrate for the THAP domain. Perhaps this approach can be applied globally to achieve a final tally of TE's contribution to proteomes. The authors also hint at a second method: the integration of the 7 sequence comparison methods to increase confidence in TE exaptation predictions. The authors show a checkered pattern of success and failure of the 7 methods to detect previously identified TE-associated proteins. From one aspect it is interesting to ask if the specific failures of some of the programs can be overcome by tuning the specific search parameter. And from another aspect, could this positive control set and the pattern of successes across the different methods be used to learn how the different methods complement each other? This seems to be especially in demand given the very limited amount of overlap between many of the methods. Such integration may lead to a more accurate list of TE exaptations.

**Authors' response: ***Dr. Yanai suggests an interesting point about the use of a combined search program approach and the adjustment of search parameters for more accurate identification of TE exaptations. This is a good idea, although a larger positive control set may be needed in order to get more information on TE exaptation signals. Several factors (e.g. age of TEs) that can affect search parameters have to be considered. As we show in *Table 6, *SETMAR, which represents an evolutionarily recent TE-CDS exaptation event that occurred during the primate radiation, maintains very strong signal that could be detected by any program we used. The ancient TE-CDSs do not retain DNA sequence similarity to the TEs from which they are derived and complementary methods will be needed to capture all such events. Indeed, there is no one method that captures all of the events, and it is difficult to appreciate exactly which combination of search techniques would be needed to ensure maximum coverage. In general, the more methods that can be used the better. Different methods will be more-or-less effective according to the type (e.g. element family and/or age) of exaptation event that is being evaluated and the same probably holds for the tuning of specific search parameters (e.g. e-value thresholds)*.

Finally, the notion that many genes contain TE's that are not translated leads to the interesting question of how the organism protects itself against disruption by TE insertions. Surprisingly TE's may not promote creativity at the protein level but instead creativity at the level of new mechanisms to curb the disruption caused by proliferating selfish elements.

**Authors' response: ***Dr. Yanai raises a good question about the tolerance to random mutation of low coding potential TE-derived coding sequences. As we proposed in the conclusion section, although lacking protein coding potential, these TE-derived exons may provide other functional roles in the genome such as gene regulation. TE sequences can be a rich source for natural antisense transcripts that regulate gene expression of many sense targets leading to the complex regulatory networks in the genome. This could possibly be the reason that untranslated TE-exons are preserved by natural selection. Furthermore, these exonized anti-sense regulators could play a role in post-transcriptional suppression of TE sequences throughout the genome and thereby represent a mechanism to "curb the disruption caused by proliferating selfish elements" as Dr. Yanai stated. While we are actively working on these ideas, much more work needs to be done to gain a better understanding of this phenomenon*.

### Reviewers' report 2

Kateryna D. Makova, Pennsylvania State University and Melissa Wilson, Pennsylvania State University (nominated by Kateryna D. Makova)

The manuscript by Piriyapongsa et al. provides an investigation of a very interesting and recently debated issue, i.e. how much transposable elements contribute to protein-coding genes. The authors discuss previous research on this topic and provide a thorough analysis to reach their final conclusions. The manuscript is well written and will be of great interest to a broad audience. We have just a few minor suggestions for the authors to consider.

1. It is clear from table 4 that PDB and Swiss-Prot contain very different protein sets. We wonder how much these two sets overlap, is one more abundant for some species than the other, and how this disparity might affect the results.

**Authors' response: ***The PDB and Swiss-Prot proteins are indeed quite different and that is one of the reasons that we analyzed these two data sets separately. The results are shown individually for PDB and Swiss-Prot proteins. We have checked for the overlap between PDB and Swiss-Prot data sets using BLAST program. 52.60% of Swiss-Prot protein (CDS) sequences have at least one PDB CDS hit with e-value <= 0.001. 72.07% of Swiss-Prot protein sequences have at least one PDB protein hit with e-value <= 0.001. So the two sets do overlap substantially. In addition, the results of most search methods, in terms of the fraction of hits obtained, were comparable across the two data sets *(Table 1*and *Table 2). *Only the results obtained from BLASTP and BLASTX program showed markedly lower percentages of PDB hits compared to Swiss-Prot data set. The lower fraction of TE-encoded sequences in PDB data set can be due to the redundancy of protein chains in PDB entries. The HMMER search, which used PDB/Swiss-Prot proteins as query sequences, also showed the difference of hit fractions between two protein data sets, though not as clearly as BLAST results. Having said all of that, the two most relevant points for our manuscript that hold for both the PDB and Swiss-Prot sets are 1-different search methods detect largely different sets of hits as described in the manuscript and 2-the different search methods show similar relative performances in terms of selectivity and sensitivity *(Figure 1).

2. Under Results and Discussions, the first section titled, "Searching for TE-associated proteins," when discussing the BLAST searches, it might be clearer to describe the three TE databases as libraries, only because it is confusing to think of searching the CDS databases with another database.

**Authors' response: ***The sentence has been edited to read "TE sequence libraries" as suggested*.

3. How do you define selectivity?

**Authors' response: ***Selectivity is defined in our manuscript as the strength of the effect of increasingly stringent E-value thresholds on the number of hits retrieved by the different search methods. This is measured in terms of the relative number (percentage) of hits retrieved at different E-value cut-off levels*.

4. Under "Comparative analysis of cases of TE-CDS exaptation", do you mean that you had the exact same hits (same proteins) for both the PDB and Swiss-Prot using GA and TC in HMMER? Please clarify.

**Authors' response: ***We meant that the GA and TC methods yield the same number of hits when run on the same database (not between databases). This was confusing and we have modified the confusing part of the sentence to clarify it*.

5. What evidence do you have for the proposal that exonized TE sequences serve as natural anti-sense transcripts, or that they may be master regulators? Perhaps the word "hypothesize" would be better suited than "propose" in the second to last sentence of the Conclusion.

**Authors' response: ***We currently have several ongoing projects in the lab that are related to this open question. The 'proposal' that TE sequences serve as natural anti-sense transcripts is supported by some new unpublished data, but we need to evaluate this more before we can be certain. Therefore, the reviewers' point is well taken and we have changed the abstract and the conclusion to read 'hypothesize' as suggested*.

6. In figure 3, if there is enough room, it would be beneficial to have the species names spelled out completely either in the figure or in the figure legend.

**Authors' response: ***For the cellular proteins, the species names have been added to the figure as suggested*.

### Reviewer's report 3

Cedric Feschotte, University of Texas, Arlington (nominated by John M. Logsdon Jr.)

The study attempts to provide a more quantitative and realistic assessment of the contribution of TE-derived sequences to the host proteome. As the authors rightfully point out in their introduction, previous investigations of this question have used different methods, different datasets and different probabilistic cut-off, resulting in extremely different outcomes and inconsistent results. So the extent by which TE contribute to the host proteome remain an unsettled issue. The study does not offer an unambiguous answer to this issue, but it provides a clear demonstration that different computational approaches can yield extremely different results, both quantitatively and qualitatively, in terms of the number of potential TE-derived coding sequences and the categories of exaptation events. Interestingly, the authors observe that the different approaches return largely non-overlapping dataset of candidate TE-derived sequences. They conclude that it will be necessary to combine these different methods to capture the whole breadth of potential TE-derived proteins. They also show, through the example of THAP-domain proteins, that phylogenetic analyses can bring further support to the claim that some host-encoded protein domains are derived from TE-encoded domains rather than vice-versa.

The second part of the study explores another outstanding question: are TE-derived exons (i.e. those with unambiguous TE origin and experimentally-supported transcription) effectively translated into functional proteins? The authors approach this question through a comparison of the coding potential of TE-derived exons and non-TE derived exons based on a probabilistic assessment of codon- and GC-biases typically produced by protein-coding sequences. The results show that the dataset of TE-derived exons examined have a dramatically different codon and GC signatures than non TE-derived exons, which the authors interpret as a reduced probability to be protein-coding sequences.

Overall, I found that this is a valuable study in that it provides a scholarly effort to assess and quantify the biases that can be introduced when different sequence similarity-search methods are used to address what seems to be the same question. The results provide an immediate explanation for the discrepancies in the degree of TE exaptation into coding sequences published previously by different groups.

(1) My major concern is that the authors have intermingled and often lumped two very different categories of TE exaptation events, which I believe should be kept and treated separately. The first type of event, which I would refer as 'domestication' is when a coding sequence previously encoded by a TE is recycled or exapted toward a cellular function. The classic examples would be RAG1 or the transposase domain of SETMAR. The second type of exaptation events, which perhaps could be referred as to 'exonization' is when a TE sequence that was previously non-coding becomes part of a new coding sequence (e.g. Lev-Maor et al. Science 2003; Krull et al. 2005). The classic example here would be exons derived from SINEs, such as Alu, which themselves originally derive from functional non-coding RNA (generally tRNA or, in the case of Alu, 7SL RNA). Intuitively, domestication events seem less dubious as to their coding potential than exonization events, because they only require that the ancestral TE coding sequence be maintained (or slightly modified) to be potentially adaptive. In contrast, exonization events require a seemingly more fortuitous evolutionary scenario to become adaptive at the protein level, such as 'de novo' emergence of a new functional domain, separation or disruption of an existing functional domain by a loop, etc... Thus, one might view exonization events as being either initially neutral and/or as having a regulatory function at the RNA level (as the authors suggest in their conclusion, see below for more discussion of this hyptothesis). Thus the adaptive value of exonization events is generally subject to more debate than domestication events. I believe treating the two categories separately will clarify and improve the quality of the study.

For example, once these two types of events are separated, it becomes evident that profile-based searches, which use a collection of established TE coding domains (as found in Pfam) as queries, can only detect domestication events, not exonization events. This simple observation can largely explain the discrepancies (Table 1) in the amount and nature of TE exaptation detected via profile-based searches and the BLAST-based searches, which use as queries all the known repeat consensus sequences, either as DNA or as translated queries (blastn or tblastx, respectively). Thus, I am not sure it really makes sense to compare side-by-side these approaches as they use different query sets and it is readily predictable that they will not yield the same results.

The comparison of profile/HMM searches with tblastn or blastp searches, which use predicted TE-encoded proteins as queries instead of the entire TE database, seems more appropriate. However, here again the query sets used in the two types of methods are not directly comparable: TE Pfam domains are generally well conserved domains often validated by biochemical/structural analyses and the resulting HMM profiles are based on multiple alignments of a broad diversity of TE and/or viral proteins. Thus the same 'consensus' HMM profile encompasses generally a very wide diversity of TEs (often an entire superfamily or class of elements, e.g. reverse transcriptase). In addition, several TE domains are not characterized and represented in conserved domain databases, so there is no derived HMM profiles for those. In contrast, predicted TE-encoded proteins (which were used as queries in tblastn and blastp searches) are not limited to characterized/conserved domains and they are more representative of the diversity of the coding potential of TEs. Thus, it is a richer source of queries to potentially detect domestication events, but the approach is hampered by the overall lack of sensitivity of blast algorithms. Conversely, the sensitivity of HMM searches is notoriously better, but since the queries are less representative of the coding capacity of TEs, the absolute number of highly significant hits tend to be lower than with blast-based methods. Consequently, I suspect that HMM searches are able to uncover more remote similarities and yield hits not detected by blastp/tblastn, while the latter searches will reveal more closely related hits and hits with TE domains not represented in the queries of the HMM searches. This can explain, in part, the lack of overlap between the methods emphasized by the authors. In conclusion, even though the authors have 'fixed' the sequence space that they explore (PDB/Swiss Prot database, a biologically reasonable compromise compared to previous studies), they are not really able to 'fix' the query sets used in the different methods. Thus, a direct comparison of the outcomes produced by these different methods may be misleading.

In my opinion, it was inherently predictable that the different methods will not yield the same datasets. For example, it is clear that if one wants to identify exonization events (as I defined above), HMM profiling, tblastn, or blastp searches are not the way to go. So, I would disagree with the conclusion (see abstract) that "profile-based methods is the most appropriate tool for detecting TE-CDS associations". It really depends what type of exaptation events you're trying to detect. The problem with the way the ms is written is that it seems that all of these methods may be used to detect the same type of events, broadly defined as TE-derived coding sequences. I think the authors should separate more clearly the two types of exaptation events and the tools that are appropriate to detect one or the other.

***Authors' response: ****The reviewer raises an important point that we have tried to address in our analysis, namely the evolutionary fate of different kinds of TE sequences that become incorporated into the mRNAs of host genes. There is indeed good evidence to suggest that different classes of TEs, i.e. those with protein coding versus those with non-protein coding sequences, may have different evolutionary fates once incorporated into the mRNAs of host (cellular) genes. This notion is well supported by *Tables 7 &8*along with *Figures 4, 5*and Supplementary Figure 1 from our manuscript. However, there are some epistemological and related terminology issues that need to be cleared up in regard to the distinction proposed by Dr. Feschotte between 'domestication' and 'exonization' events. Molecular domestication, a phrase introduced into the biological lexicon by Wolfgang J. Miller, is a generic term that refers to any number of different events whereby a formerly selfish TE sequence becomes domesticated to play some role for its host. Similarly, the term exaptation, introduced by Stephen Jay Gould and Elisabeth Vrba, refers to any biological feature that evolved for one purpose but then acquires a new function for which it was not originally adapted, such as a TE sequence that becomes a functional part of a host gene. The relevant point here is that domestication and/or exaptation events can be deduced to have occurred irrespective of the particular mechanism by which they arose or their mode of action for that matter. For instance, TE sequences can be domesticated to serve as protein coding sequences but they can also be domesticated to serve as regulatory sequences. Exonization, on the other hand, simply refers to the actual mutational event whereby a TE sequence acquires the genomic location and signals that cause it to be captured as an exon in a host gene's mRNA. The question we are addressing here is what happens after exonization. There can be several fates for exonized TEs: 1-they can be deleterious and selected against, 2-they can be neutral and drift to fixation or be lost, or 3-they can be beneficial and consequently fixed by selection. For the last category, the molecular function that leads to a benefit for the host and subsequent adaptive fixation can be different: it could be a role as a protein coding sequence or it could be a regulatory role such as we propose for anti-sense regulation. We are in full agreement with the reviewer that different classes of TEs probably have different propensities to be domesticated as protein coding versus regulatory sequences. However, dividing these events into exonization versus domestication does not accurately capture the nuances of the biological events that we are trying to analyze. The exonization-domestication dichotomy also obscures an important distinction between mutational events that originate genomic changes and the evolutionary events that follow. Nevertheless, Dr. Feschotte makes a good point about the importance of clearing up the difference between coding versus non-coding TE sequences being exonized and their subsequent fates. In order to clarify this, we describe how previous authors have made this distinction and how it relates to our study in the introduction, results and conclusion sections. In addition, we have added an analysis of the protein coding potential of well-characterized TE-CDS exaptation events shown in *Table 6, *all of which represent cases where TE coding sequences were exonized and subsequently exapted. We also specifically delineate the different datasets used in the different parts of the manuscript*.

*While Dr. Feschotte predicted that the different search approaches used would turn up almost entirely different sets of results, we did not anticipate the level of dissonance observed. In addition, this point has not been sufficiently appreciated in the literature to date, and this is precisely one of the things that we were trying to evaluate. The other issues that the reviewer points out about the different query versus search sets along with the different affinities of the search methods used are quite correct and of course unavoidable given the use of different methods in our study. However, we emphasize again, and also mention in the paper, that the effect of these differences on the search for TE-derived protein coding sequences was precisely what we were trying to evaluate. One of the confounding results of this study (see response to Reviewer's report 1) is that it is still not apparent exactly which methods (or search parameters) will be best suited to which kinds of events. For this reason, we conclude that the most rigorous searches of TE-CDS exaptation events should include as many different and complementary search methods as is possible*.

(2) A related issue is that the first part of the ms puts more emphasis on domesticated TE domains (e.g. ability to detect known domesticated proteins, example of the THAP domain), while the second part of the ms addresses mostly the coding potential of exonized TEs. But, without careful consideration of the datasets examined and of the methods to assemble the datasets, the reader might be tempted to conclude that the conclusions of the second part apply to all TE-derived coding sequences, while I suspect they only apply to exonized TEs sensu stricto. Indeed, the dataset used in the second part was obtained by intersecting the coordinates of human CDS and those of annotated TEs (i.e. identified by RepeatMasker). For having personally worked with this same dataset, I recall that it consists essentially of short exonized TE fragments that were not ancestrally coding (Alu, MIRs, various MERs...etc) or when they were, the present coding sequence is not derived from the coding part of the element or from the ancestral reading frame (e.g. ancient L1, L2...etc). Most of the known cases of domesticated TEs are not captured by this procedure (e.g. CENP-B, RAG1, ZBED) because they occurred too long ago (as shown in Tables 5 and 6, and discussed in the text earlier, these are not detectable by Repeatmasker because there is not enough similarity between human TEs and the ones that were originally domesticated). I think this point is important and should be made clearer in the text. It might be enlightening to analyze separately the coding potential of domesticated TE domains from the exonized non-coding TEs as the authors did by analyzing separately Alu-derived exons. I suspect they would find a significant difference between the two different categories of exaptation events in terms of their coding potential.

**Authors' response: ***Here, we were trying to evaluate the coding potential of TE sequences that have been exonized into human genes but where we do not know if they are actual protein coding sequences or not. The point about short fragments is well taken but these were eliminated prior to analysis as described in the manuscript. We did try to analyze the coding potential of different classes separately as the reviewer suggests but the relatively low numbers of long-enough fragments did not afford enough resolution to our analysis. There are 160 TE-derived fragments with the minimum length of 100 nt required for GeneMark analysis and when we attempted to divide these into different classes we lost resolution. Thus, there is not sufficient data to do the comparative analysis suggested by the reviewer. Nevertheless, Dr. Feschotte raises a valuable point about the need to clarify the difference between RepeatMasker identified TE-derived exons and the exons derived from more ancient TE-CDS exaptation events. We have added a new paragraph, and a new analysis, to the manuscript to address this point. In order to evaluate the protein coding potential of known TE-CDSs, we have run GeneMark on the previously identified TE-CDS events listed in *Table 6. *These sequences do have strong protein coding potential and in this sense stand out from the abundant exonized Alu (and other) elements that we analyzed*.

(3) In the second part of the ms, the authors show convincingly that TE-derived exons display a significantly different 'codon' (as measured by GeneMark) and GC bias than non TE-derived exons. They interpret these differences as evidence against the coding potential of TE-derived exons. Alternatively, this difference might also reflect the relatively more recent evolutionary origin of TE exons compared to non-TE exons. This could also reflect the fact that most TE-derived sequences examined were previously non-coding (this is certainly true for Alu-derived exons) and had a biased nucleotide composition (Alus for example are GC-rich) prior to exonization. It could be that the codon/GC biases characteristics of non-TE exons require the passage of relatively long evolutionary time to become apparent. So, if the TE-derived sequences are evolutionary recent (Alu for example is a primate-specific family), they might simply not (yet) exhibit these biases. It would be interesting to see whether more ancient TE-derived exons (e.g. derived from MIRs, L2...) have a different codon/GC signature than recently derived TE exons (e.g. those derived from recent L1 or Alus). The age of exonized TEs could be estimated easily based on their presence/absence in other mammals (mouse for example) using the comparative genomics track of the UCSC Genome Browser. If broadly conserved TE-derived exons are still found to display a strikingly different codon/GC signature than non-TE exons (but similar to those of recent Alus), then it will strengthen the argument that they have reduced or no coding potential (as suggested in the last paragraph of the Conclusion).

**Authors' response: ***One of the challenges of the probabilistic GeneMark analysis is the relatively long TE-exon sequences needed together with the high numbers needed to distinguish between distributions. As mentioned in the response to point #2, there are simply not enough data points to divide the TE-derived exons into the number of different classes needed to evaluate different age classes of elements. While this is the case, the reviewers point about the relative ages of the elements is a valid one and the fact that the Alus, for which there are high enough numbers to evaluate separately, are a relatively evolutionarily young set of elements suggests that the low apparent coding potential may indeed reflect the fact that there has not been enough evolutionary time elapsed since exonization to change the structure of the coding regions. We have added a sentence to reflect this fact to the manuscript*.

(4) Section "comparative analysis" (Table 4):

Here, the authors should clarify what they mean by "synthetic constructs". They should also explain how they distinguish between specific and non-specific TE domains. I concur that it is often difficult to infer whether a given domain is of TE origin or was acquired from the host by TEs. They use the example of the RNase H fold, but it might not be the best example because this fold is also related to the DDE core of retroviral integrases and TPases. Discarding systematically hits to Rnase H might exclude a number of bonafide TPase or IN-derived proteins. Non-specific domains would require closer inspection before being disregarded as false positives. The level of similarity (reflected by e-values) could be used as a guide. Similarly, I am not convinced that viral-like domains should be systematically regarded as unreliable cases of domesticated TE coding sequences because many robust cases of TE-derived proteins might actually fall within this category (for example gag-like proteins derived from extinct gypsy-like elements from vertebrates – see Campillos et al. TIG 2006).

**Authors' response: ***Synthetic constructs are artificial, i.e. man-made or modified sequences, such as vectors or sequence fragments (not the entire native protein), and this has now been clarified for the reader in the manuscript. As for the non-specific category, we agree completely with the reviewer that these cases should not necessarily be dismissed out-of-hand. However, here we have tried to be as conservative as possible in order to focus on the most reliable cases, and this is also explained for the reader where we mention that it is possible that these cases represent ancient TE-CDS exaptation events. The viral proteins are also potentially interesting, but we eliminate them from consideration since our own inspection of this class revealed that the vast majority are bona fide viral proteins, many of which are grouped together with TE-derived proteins when forming Pfam-models. For instance, none of the viral proteins in *Table 4*are likely to correspond to TE-derived proteins*.

(5) I was perplexed by the section 'case studies of known TE-derived proteins' (Table 6). First, the authors use RAG2 as a known case of transposase domestication. However, to my knowledge, there is no evidence that RAG2 is related to a transposase. Both RAG1 and RAG2 are required for V(D)J recombination, but only RAG1 contains the DDE catalytic core and is affiliated to transposases (Transib; see Kapitonov & Jurka 2005). The origin of RAG2 remains undetermined. So, it is not surprising that RAG2 is not detected as TE-derived by any of the Blast or RM methods. However, I found it extremely surprising that it is detected using HMM. This would be a new and exciting finding, which if confirmed, would require further explanation. In particular, I was wondering what Pfam entry hit with RAG2? Conversely, I am surprised that HMM did not detect RAG1, because the link with DDE recombinases is very well established and the RNAseH/DDE fold of RAG1 is well conserved. This is precisely the type of distant relationship that I would expect HMM to be superior at detecting than Blast-based methods. Are the authors sure they did not swap the HMM results of RAG1 and RAG2 in Table 6? In this case, the conclusion that "the only way to detect all of these known cases would be to used a combined strategy based on both profile and translated-blast strategies" does not hold anymore as the two strategies will detect all of them. Plus, if we exclude the case of RAG2, it appears that translated blast strategies will detect sufficient to detect all of them.

**Authors' response: ***This was a mistake in our analysis and we are grateful to Dr. Feschotte for pointing this out. Accordingly, the RAG2 example has been eliminated from the manuscript. A transposase evolutionary origin of RAG2 was proposed in the literature based on biochemical/functional analysis – but this could well be due to the fact that it acts in a complex with RAG1 as suggested. On the other hand, RAG1 was also identified as being related to transposase through biochemical/functional studies originally but then it was recently identified as having sequence similarity to transposases. However, the results we present for RAG1 were not a case of switching RAG1 and RAG2, so the result of RAG1 not being detected with the HMM search holds up. In any case, removing RAG2 does mean that the translated BLAST searches are sufficient to detect all cases as Dr. Feschotte states. Thus, the fact that the different search methods are complementary is better exemplified by the data in *Tables 1*and *2.

(6) I was surprised that the authors did not mention the utility of selection analysis on codons (Ka/Ks) in the assessment of the coding potential of TE-derived coding domains. This type of analysis remains one of the most reliable (although indirect) methods to assess whether a given transcribed sequence is actually being translated into a functional protein. In particular, evidence of purifying selection in the form of Ka/Ks ratio significantly lower than 1 in TE-derived exons can be taken as a strong indication that they encode functional coding sequences. The method is also powerful because it allows discriminating between domesticated proteins and TE-encoded proteins as the latter tend to evolve neutrally (a good example is provided by the study of domesticated Drosophila PIF-like proteins by Casola et al. MBE 2007).

**Authors' response: ***We did attempt Ka/Ks analysis, but since many of the TE-derived exons are relatively young, there was not enough data to perform a systematic study of Ka/Ks. We agree that this technique is quite powerful and reliable, in principle, for evaluating individual cases of TE-CDS exaptation. The problem is that you need a number of substitutions in order to get good statistical resolution between Ka and Ks but not so many as to saturate Ks. For most cases we looked at, there were simply not enough publicly available orthologous, and long enough, TE-exon regions available for analysis. In fact, this is one of the very reasons that we attempted the relatively novel codon based analyses described to evaluate the coding potential of TE-derived exons. The fact that these probabilistic methods read along the length of the entire TE-derived sequence, as opposed to simply evaluating a handful of substitutions between sequences, gives them a relatively higher degree of resolution based on the amount of data (sites) that they evaluate. Having said that, even these methods provided challenges in terms of resolution as described in the responses to points #2 and #3. The distinction between Ka/Ks and the probabilistic approaches we take were not raised in the manuscript because we did not intend to debate the relative merits of the different classes of codon based analysis*.

(7) In their conclusion paragraph and in the abstract, the authors propose that many of the exonized TE sequences have a regulatory function at the RNA level. They hypothesized that they "serve as natural antisense transcripts". I think the authors should provide a more explicit mechanism and at least some preliminary data supporting this model. Are they suggesting that the repeats provide promoters driving anti-sense transcription of the flanking exons? Or are they suggesting that the TE-derived exons are themselves subject to RNA interference by pairing of anti-sense transcripts produced from related repeat copies elsewhere in the genome? It would strengthen the model if the authors could provide data or published information supporting either of these models. Another model is that TE-derived exons are subject to alternative splicing and that TE-containing spliced variants are subject to non-sense mediated decay (NMD), thereby participating to post-transcriptional gene regulation. This hypothesis is supported by the observation that many TE-derived exons are indeed alternatively spliced (e.g. Lev-Maor et al. Science 2003; Krull et al. Genome Res 2007). In addition, several instances of highly-conserved TE-containing cassette exons introducing premature stop codons have been recently shown to trigger NMD (see Mendell et al. Nat Genet 2004; Bejerano et al. Nature 2006; Mola et al. J Mol Biol 2007; Ni et al. Genes Dev 2007). These observations seem highly relevant to the discussion.

**Authors' response: ***We are currently actively working on this exact question, but the data are not ready to be presented in manuscript form. The same issue was raised in Reviewers' report 2. Since we are not yet ready to present such data, we have re-worded the abstract and the conclusion to emphasize that the regulatory function of TE-derived exons at the RNA level is a hypothesis. We agree with the points the reviewer makes about the different possible models that could be involved and appreciate the references provided. The observations are indeed 'highly relevant' as Dr. Feschotte points out, and clarification would surely strengthen the model. However, such clarification will have to wait for another day and another study when the data permit*.

(8) The case of the THAP domain has been discussed extensively by Quesneville et al. (MBE 2005). This ref should be included.

**Authors' response: ***Thanks for pointing this out; we have added this reference to the paper along with a discussion of the relevance of their findings to our own*.

## Supplementary Material

Additional file 1 TE-associated protein domains. This file contains the list of 124 Pfam domains associated with TEs. Click here for file

Additional file 2 The GC composition of Alu-derived gene fragments. Scatter plots of %G+C of second (GC2) versus third (GC3) codon positions for Alu-derived gene fragments (pink) non Alu TE-derived gene fragments (red) and non-TE associated genes (green) are shown along with linear regression trends and confidence interval. Click here for file
