# Supplementary material for: Evaluating the protein coding potential of exonized transposable element sequences
Source: Biol Direct. 2007 Nov 26;2:31. doi: 10.1186/1745-6150-2-31 (PMC2203978; doi:10.1186/1745-6150-2-31)
Supplement: Additional file 1 — TE-associated protein domains. This file contains the list of 124 Pfam domains associated with TEs. [file 1745-6150-2-31-S1.pdf]

Supplementary Table 1 - List of 124 TE-associated Pfam protein domains

Accession	ID	Description
PF00075	RnaseH	RNase H
PF00077	RVP	Retroviral aspartyl protease
PF00078	RVT_1	Reverse transcriptase (RNA-dependent DNA polymerase)
PF00098	zf-CCHC	Zinc knuckle
PF00424	REV	REV protein (anti-repression trans-activator protein)
PF00429	TLV_coat	ENV polyprotein (coat polyprotein)
PF00469	F-protein	Negative factor, (F-Protein) or Nef
PF00516	GP120	Envelope glycoprotein GP120
PF00517	GP41	Envelope Polyprotein GP41
PF00522	VPR	VPR/VPX protein
PF00539	Tat	Transactivating regulatory protein (Tat)
PF00540	Gag_p17	gag gene protein p17 (matrix protein)
PF00552	Integrase	Integrase DNA binding domain
PF00558	Vpu	Vpu protein
PF00559	Vif	Retroviral Vif (Viral infectivity) protein
PF00589	Phge_integrase	Phage integrase family
PF00607	Gag_p24	gag gene protein p24 (core nucleocapsid protein)
PF00665	rve	Integrase core domain
PF00692	dUTPase	dUTPase
PF00872	Transposase_mut	Transposase, Mutator family
PF00906	Hepatitis_core	Hepatitis core antigen
PF00971	EIAV_GP90	EIAV coat protein, gp90
PF00979	Reovirus_cap	Reovirus outer capsid protein, Sigma 3
PF01021	TYA	TYA transposon protein
PF01045	EIAV_GP45	EIAV glycoprotein, gp45
PF01054	MMTV_SAg	Mouse mammary tumour virus superantigen
PF01140	Gag_MA	Matrix protein (MA), p15
PF01141	Gag_p12	Gag polyprotein, inner coat protein p12
PF01359	Transposase_1	Transposase
PF01385	Transposase_2	Probable transposase
PF01498	Transposase_5	Transposase
PF01526	Transposase_7	Transposase
PF01527	Transposase_8	Transposase
PF01548	Transposase_9	Transposase
PF01609	Transposase_11	Transposase DDE domain
PF01610	Transposase_12	Transposase
PF01695	IstB	IstB-like ATP binding protein
PF01710	Transposase_14	Transposase
PF01797	Transposase_17	Transposase IS200 like
PF02022	Integrase_Zn	Integrase Zinc binding domain
PF02093	Gag_p30	Gag P30 core shell protein
PF02228	Gag_p19	Major core protein p19
PF02281	Transposase_Tn5	Transposase Tn5 dimerisation domain
PF02316	Mu_DNA_bind	Mu DNA-binding domain
PF02337	Gag_p10	Retroviral GAG p10 protein
PF02371	Transposase_20	Transposase IS116/IS110/IS902 family
PF02411	MerT	MerT mercuric transport protein
PF02720	DUF222	Domain of unknown function DUF222
PF02813	Retro_M	Retroviral M domain
PF02892	zf-BED	BED zinc finger
PF02914	Mu_transposase	Bacteriophage Mu transposase
PF02920	Integrase_DNA	DNA binding domain of tn916 integrase
PF02959	Tax	HTLV Tax

PF02992	Transposase_21	Transposase family tnp2
PF02994	Transposase_22	L1 transposable element
PF02998	Lentiviral_Tat	Lentiviral Tat protein
PF03004	Transposase_24	Plant transposase (Ptta/En/Spm family)
PF03017	Transposase_23	TNP1/EN/SPM transposase
PF03050	Transposase_25	Transposase IS66 family
PF03056	GP36	Env gp36 protein (HERV/MMTV type)
PF03078	ATHILA	ATHILA ORF-1 family
PF03108	MuDR	MuDR family transposase
PF03184	DDE	DDE superfamily endonuclease
PF03221	Transposase_Tc5	Tc5 transposase
PF03274	Foamy_BEL	Foamy virus BEL 1/2 protein
PF03276	Gag_spuma	Spumavirus gag protein
PF03400	Transposase_27	IS1 transposase
PF03408	Foamy_virus_ENV	Foamy virus envelope protein
PF03539	Spuma_A9PTase	Spumavirus aspartic protease (A9)
PF03708	Avian_gp85	Avian retrovirus envelope protein, gp85
PF03716	WCCH	WCCH motif
PF03732	Retrotrans_gag	Retrotransposon gag protein
PF03811	Ins_element1	Insertion element protein
PF04094	DUF390	Protein of unknown function (DUF390)
PF04160	Borrelia_orfX	Orf-X protein
PF04195	Transposase_28	Putative gypsy type transposon
PF04218	CENP-B_N	CENP-B N-terminal DNA-binding domain
PF04236	Transp_Tc5_C	Tc5 transposase C-terminal domain
PF04582	Reo_sigmaC	Reovirus sigma C capsid protein
PF04693	Transposase_29	Archaeal putative transposase ISC1217
PF04740	Transposase_30	Bacillus transposase protein
PF04754	Transposase_31	Putative transposase, YhgA-like
PF04827	Plant_tran	Plant transposon protein
PF04937	DUF659	Protein of unknown function (DUF 659)
PF04986	Transposase_32	Putative transposase
PF05052	MerE	MerE protein
PF05344	DUF746	Domain of Unknown Function (DUF746)
PF05380	Peptidase_A17	Pao retrotransposon peptidase
PF05399	EVI2A	Ectropic viral integration site 2A protein (EVI2A)
PF05457	Transposase_33	Sulfolobus transposase
PF05485	THAP	THAP domain
PF05598	DUF772	Sulfolobus solfataricus protein of unknown function (DUF772)
PF05599	Deltaretro_Tax	Deltaretrovirus Tax protein
PF05621	TniB	Bacterial TniB protein
PF05699	hATC	hAT family dimerisation domain
PF05717	Transposase_34	IS66 Orf2 like protein
PF05754	DUF834	Domain of unknown function (DUF834)
PF05840	Phage_GPA	Bacteriophage replication gene A protein (GPA)
PF05851	Lentivirus_VIF	Lentivirus virion infectivity factor (VIF)
PF05858	BIV_Env	Bovine immunodeficiency virus surface envelope protein (ENV)
PF05928	Zea_mays_MuDR	Zea mays MURB-like protein (MuDR)
PF06527	TniQ	TniQ
PF06815	RVT_connect	Reverse transcriptase connection domain
PF06817	RVT_thumb	Reverse transcriptase thumb domain
PF07253	Gypsy	Gypsy protein
PF07282	Transposase_35	Putative transposase DNA-binding domain
PF07567	zf-C2HC_plant	Protein of unknown function, DUF1544
PF07572	BCNT	Bucentaur or craniofacial development
PF07592	Transposase_36	Rhodopirellula transposase
PF07727	RVT_2	Reverse transcriptase (RNA-dependent DNA polymerase)
PF07999	RHSP	Retrotransposon hot spot protein

PF08284	RVP_2	Retroviral aspartyl protease
PF08333	DUF1725	Protein of unknown function (DUF1725)
PF08483	IstB_N	IstB-like ATP binding N-terminal
PF08705	Gag_p6	Gag protein p6
PF08721	TnsA_C	TnsA endonuclease C terminal
PF08722	TnsA_N	TnsA endonuclease N terminal
PF08723	Gag_p15	Gag protein p15
PF09035	Tn916-Xis	Excisionase from transposon Tn916
PF09039	Mu_I-gamma	Mu DNA binding, I gamma subdomain
PF09077	Phage-MuB_C	Mu B transposition protein, C terminal
PF09293	RNaseH_C	T4 RNase H, C terminal
PF09299	Mu-transpos_C	Mu transposase, C-terminal
PF09322	DUF1979	Domain of unknown function (DUF1979)
