# Supplementary material for: Evaluating the protein coding potential of exonized transposable element sequences
Source: Biol Direct. 2007 Nov 26;2:31. doi: 10.1186/1745-6150-2-31 (PMC2203978; doi:10.1186/1745-6150-2-31)
Supplement: Additional file 2 — The GC composition of Alu-derived gene fragments. Scatter plots of %G+C of second (GC2) versus third (GC3) codon positions for Alu-derived gene fragments (pink) non Alu TE-derived gene fragments (red) and non-TE associated genes (green) are shown along with linear regression trends and confidence interval. [file 1745-6150-2-31-S2.pdf]

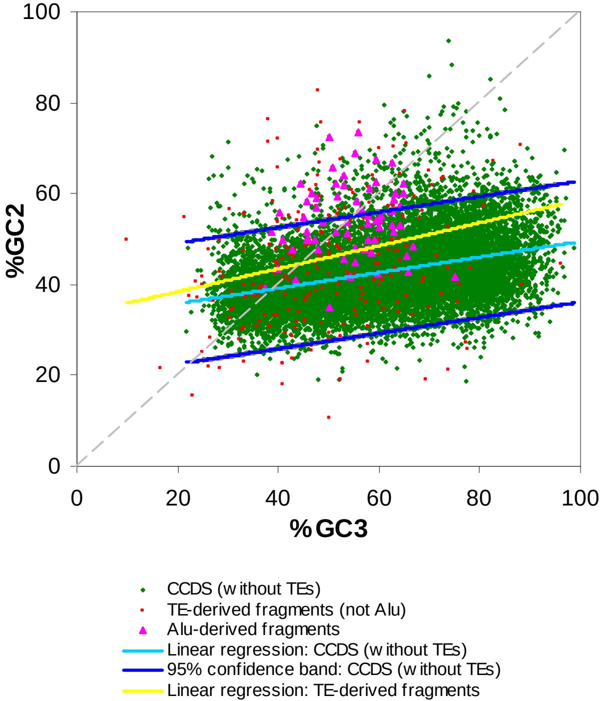

100
80
60
40
20
0
%GC2
0
20
40
60
80
100
%GC3
CCDS (without TEs)
TE-derived fragments (not Alu)
Alu-derived fragments
Linear regression: CCDS (without TEs)
95% confidence band: CCDS (without TEs)
Linear regression: TE-derived fragments

Supp Fig 1
